# Orange County, California COVID-19 Vaccine Equity Best Practices Checklist: A Community-Centered Call to Action for Equitable Vaccination Practices

**DOI:** 10.1089/heq.2021.0048

**Published:** 2022-01-17

**Authors:** Kameko J. Washburn, Alana M.W. LeBrón, Abigail S. Reyes, Isabel Becerra, America Bracho, Ellen Ahn, Ana Siria Urzúa, Mary Anne Foo, Salvador Zárate, Sora Park Tanjasiri, Bernadette Boden-Albala

**Affiliations:** ^1^ Department of Health, Society, and Behavior, Program in Public Health, Susan and Henry Samueli College of Health Sciences, University of California, Irvine, Irvine, California, USA.; ^2^ Department of Chicano/Latino Studies, School of Social Sciences, University of California, Irvine, Irvine, California, USA.; ^3^ Community Resilience Projects, University of California, Irvine, Irvine, California, USA.; ^4^ Coalition of Orange County Community Health Centers, Santa Ana, California, USA.; ^5^ Latino Health Access, Santa Ana, California, USA.; ^6^ Korean Community Services, Buena Park, California, USA.; ^7^ Cooperación Santa Ana, Santa Ana, California, USA.; ^8^ Orange County Asian and Pacific Islander Community Alliance, Garden Grove, California, USA.; ^9^ Department of Anthropology, School of Social Sciences, University of California, Irvine, Irvine, California, USA.; ^10^ Department of Epidemiology, Program in Public Health, Susan and Henry Samueli College of Health Sciences, University of California, Irvine, Irvine, California, USA.

**Keywords:** COVID-19, vaccine equity, health equity, community–academic partnership, community centered

## Abstract

**Introduction:** The coronavirus disease 2019 (COVID-19) pandemic has exacerbated longstanding inequities throughout the United States, disproportionately concentrating adverse social, economic, and health-related outcomes among low-income communities and communities of color. Inequitable distribution, prioritization, and uptake of COVID-19 vaccines due to systemic and organizational barriers add to these disproportionate impacts across the United States. Similar patterns have been observed within Orange County, California (OC).

**Methods:** In response to COVID-19 vaccine inequities unfolding locally, the Orange County Health Equity COVID-19 community–academic partnership generated a tool to guide a more equitable vaccine approach. Contents of the OC vaccine equity best practices checklist emerged through synthesis of community-level knowledge about vaccine inequities, literature regarding equitable vaccination considerations, and practice-based health equity guides. We combined into a memo: the checklist, a written explanation of its goals and origins, and three specific action steps meant to further strengthen the focus on vaccine equity. The memo was endorsed by partnership members and distributed to county officials.

**Discussion:** Since the initial composition of the checklist, the local vaccine distribution approach has shifted, suggesting that equitable pandemic responses require continual re-evaluation of local needs and adjustments to recommendations as new information emerges. To understand and address structural changes needed to reduce racial and socioeconomic inequities exacerbated by the pandemic, authentic partnerships between community, academic, and public health practice partners are necessary.

**Conclusion:** As we face continued COVID-19 vaccine rollout, booster vaccination, and future pandemic challenges, community knowledge and public health literature should be integrated to inform similar equity-driven strategic actions.

## Introduction

The coronavirus disease 2019 (COVID-19) pandemic has exacerbated longstanding racial, social, and economic inequities within the United States, leading to disproportionate effects among low-income communities, indigenous communities, and communities of color in terms of risk of exposure to the severe acute respiratory syndrome coronavirus 2 (SARS-CoV-2) pathogen, limited access to resources to protect against transmission, and related inequities in morbidity and mortality.^1–9^ Early doses of the vaccine were disproportionately distributed to high-income white adults.^10,11^

Inequitable distribution and prioritization of the initially limited vaccine supply, in addition to disproportionately low uptake among members of communities most affected by COVID-19 adversely affect our ability to achieve widespread immunity,^4,12–14^ and may heighten COVID-19–related health inequities.^15^ COVID-19 vaccine inequities are best understood through public health frameworks of structural and social determinants of health, fundamental causes, and racial capitalism, which illuminate the obdurate influence of systemic structural factors in maintaining and exacerbating health inequities at individual, family, community, and population levels: even as new diseases emerge and new resources are harnessed and distributed to end the pandemic.^16–19^

Hereunder, we describe the Orange County, California (OC) experience with vaccine inequities and present vaccine equity guidelines developed by the OC Health Equity COVID-19 community–academic partnership, which was formed in response to ongoing COVID-19 inequities and further harm generated by local COVID-19 response strategies. These guidelines integrate community knowledge and public health literature to inform strategic actions in equitable COVID-19 vaccine implementation at present, future COVID-19 booster vaccinations, and vaccine distribution efforts for future pandemics.

### Local context

Despite being a majority–minority county since 2004,^20,21^ racial/ethnic minority populations in OC are disproportionately affected by inequities in the social determinants of health that shape poorer health outcomes than non-Latino white counterparts.^22,23^ Given that OC is the sixth most populated county in the United States, OC's 34% Hispanic/Lation, 21.7% Asian, 0.4% Native Hawaiian and other Pacific Islander, 2.1% black or African American, and 1.0% American Indian and Alaska Native population represent >1.8 million individuals.^21^ In addition, despite OC's percentage of persons living in poverty being slightly lower than the national average (9.5% vs. 10.5%, respectively), this encompasses >300,000 individuals.^21^

During the COVID-19 pandemic, low-income communities and communities of color within OC have experienced higher rates of SARS-CoV-2 infection and COVID-19 morbidity and mortality, similar to patterns observed nationwide.^24–26^ Within California, a major factor in this higher rate of infection rests in the over-representation of low-income communities and communities of color in front-line public-facing low-wage essential jobs such as farm work, cleaning and janitorial, and food industries.^27^ This has left 5.7 million of these workers at heightened risk of COVID-19.^28^

Compounding this risk for the 19% of the state's essential workforce living in near-poverty conditions^29^ is essential workers' limited access to health services and/or sick days. The state's initial move toward age-based vaccine priority did not center vulnerable essential workers and left various community labor organizations and labor unions concerned about vaccine equity for those whose exposure is exacerbated by existing structural racial inequity and capitalist exploitation at the core of U.S. racial capital.^17,28,30–32^

Although federal organizations including the Centers for Disease Control and Prevention provided some guidance for vaccine distribution, the federal government did not establish a national infrastructure to support local distribution plans. In many cases, states have given county health departments discretion in applying vaccine eligibility categories and left distribution solutions to counties. Consequently, OC, as elsewhere, was tasked with developing and implementing its distribution plan for the COVID-19 vaccine.

Moreover, politically induced attacks on evidence-based public health efforts and the officials who promulgate them have contributed to institutional turnover and loss of longstanding public health leaders who are critical partners in resolving the COVID-19 pandemic.^33,34^ In the absence of federal and societal support for evidence-based public health practice, health departments struggled during the pandemic's first year to implement equitable COVID-19 mitigation, testing, and vaccination strategies.^12,35,36^

### Orange County health equity COVID-19 community–academic partnership

In May of 2020, the Orange County Health Equity COVID-19 community–academic partnership (see Acknowledgments section for list of partner organizations) was formed to bring together community-based health equity leaders and health equity academic partners to elevate the need for and guide local COVID-19 equity response and recovery initiatives. The partnership was formed under the belief that building local resilience in response to the pandemic is not about bouncing back to prepandemic conditions.^37^ Rather, it is about bouncing forward by centering community members' active participation in creating the OC public health and safety interventions needed to eliminate health inequities and to build health equity.^37–39^

When local actions reflect this orientation, we believe that OC will be better positioned to (1) refrain from increasing and exacerbating harm to local communities of color, indigenous communities native to the land and who have been displaced to Orange County, low-income communities that are suffering social and economic hardships, those who are homeless or facing housing insecurity, and people who are incarcerated or detained—all of whom have faced disproportionate negative impacts of COVID-19 and (2) spur and foster social cohesion, inclusion, and trust in our public health and safety pandemic responses in ways that increase the power, participation, and long-term vitality of such communities.^3,5–6,9,40–42^

Building upon our vision, members worked together in the early months of our partnership to inform strategic actions to improve equitable access to COVID-19 testing, data, and communication; support the training of contact tracers and resident-led COVID-19 response^43^; and urge the implementation and maintenance of mask mandates. As COVID-19 vaccine development rapidly progressed and the first emergency use authorization of a vaccine to combat SARS-CoV-2 was issued by the U.S. Food and Drug Administration in December 2020,^44^ we shifted our collective focus toward vaccine equity.

This shift occurred as part of our continued process of identifying priority areas in advocating for equitable COVID-19 recovery. In the following section, we describe our partnership processes and approach to advocating for equitable vaccine allocation, distribution, outreach, communication, and data monitoring through the development and dissemination of a vaccine equity tool centered around the pivotal role of community-based knowledge and localized context in shaping recommendations.

## Methods

### Partnership processes

The Orange County Health Equity COVID-19 community–academic partnership implemented several strategies to center our work around the voices of community partners while attending to potential power imbalances between academic and community partnership members. Throughout this stage of the partnership, we held biweekly meetings during which community and academic partners cocreated agendas with items suggested by community partners taking priority in terms of both order discussed and time allotted.

Depending on community partner availability and capacity to attend partnership meetings, academic partners would help to focus meetings on agenda items most relevant to those present. Academic partners' roles during these meetings centered on facilitating dialogue between community partners, listening to and identifying themes within community partners' experiences, and codeveloping action plans to address these themes. Academic partners would also connect with members who were unable to attend to share updates and create space for additional feedback.

Ultimately, academic partners aimed to support community partners' priorities—especially when navigating difficult conversations where further power imbalances may exist or when leveraging resources available through the university would be beneficial. One example of how this has been operationalized is by academic partners facilitating conversations with local policy leaders with whom community partners expressed unease around discussing relevant issues freely due to underlying political considerations that did not constrain academic voices in the same way. This partnership and vaccine equity advocacy strategy is classified as exempt by the University of California, Irvine.

### Vaccine equity advocacy

In January 2021, shortly after COVID-19 vaccine rollout began, partnership members amplified longstanding concerns regarding vaccine inequities unfolding in OC communities of color, indigenous communities, and low-income communities.

As a microcosm of trends nationwide,^12^ OC's local public health department's (Orange County Health Care Agency [OCHCA]) initial vaccine rollout efforts focused on prioritizing the vaccine to adults 65 years of age and older—regardless of race/ethnicity, socioeconomic position, or zip code^45,46^;—implemented vaccine sign-up through a mobile application that was only available in English for the first three weeks of the vaccine rollout^45,47^; and only made the vaccine available at super points of distribution (Super POD) such as Disneyland and a small private university located in an affluent region.^45,46^ This initial rollout rendered vaccination sites and vaccines accessible to more economically, technologically, and time resourced communities that were English language dominant.^45^

Although aware of vaccine shortages and OCHCA's multifaceted response efforts, partnership members emphasized the crucial need to strengthen the vaccine equity approach, with particular attention to communities experiencing the greatest negative impacts of COVID-19. Partnership members engaged OCHCA by calling for vaccine equity in spaces such as OCHCA-designated task force meetings, one-on-one discussions between county officials and partnership members, and partnership planning meetings.

**FIG. 1.**
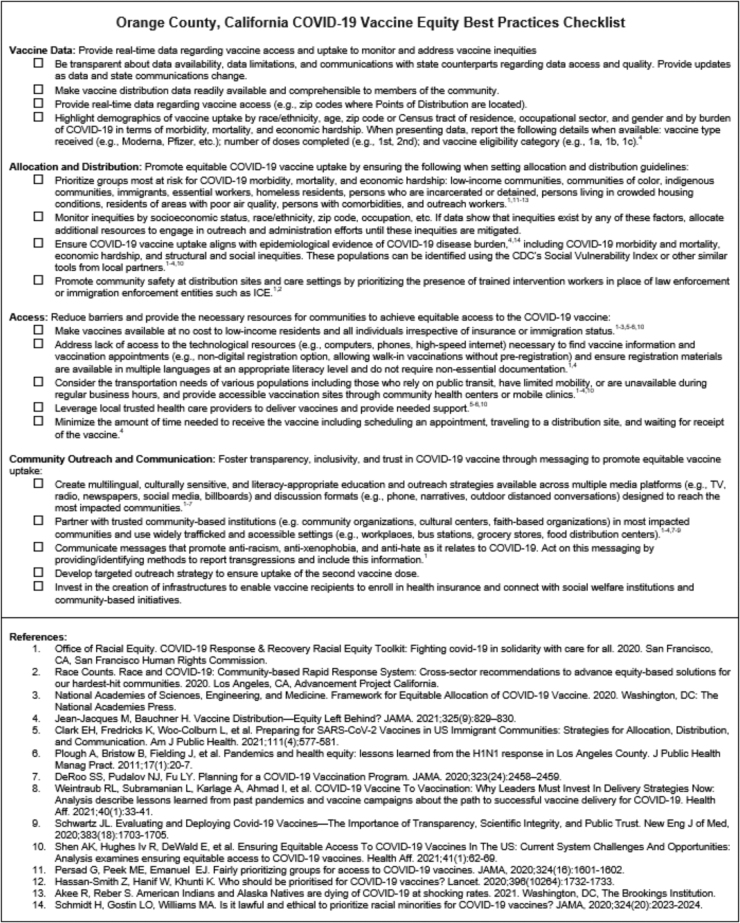
Orange County, California COVID-19 vaccine equity best practices checklist. COVID-19, coronavirus disease 2019.

After these efforts, community partners acknowledged OCHCA's progress toward improving equitable vaccine access (e.g., establishing vaccine mini-PODs), while emphasizing that more needs to be done as the populations they serve were still facing inequitable vaccine access. Community partners invited our partnership to develop the OC COVID-19 vaccine equity best practices checklist (Fig. 1) with hopes that this tool would guide a more strategic vaccine approach by strengthening policy advocacy and improving informed public discourse regarding vaccine equity in OC.

After listening carefully to the concerns raised by community partners, academic partners responded to community members' invitation and began to draft the checklist. The checklist draft underwent multiple reviews by community and academic partners through an iterative process. Thus, the final checklist was generated through the synthesis of community-level knowledge in the form of partnership member input that prioritized the perspectives of community leaders, a strategic review of the literature regarding equitable vaccine distribution considerations, and health equity guides produced by similar coalitions in other regions of California.^48–51^

## Results

### OC COVID-19 vaccine equity best practices checklist

Based on the literature and priority areas emphasized by community partners, we organized the OC COVID-19 vaccine equity best practices checklist to highlight vaccine equity best practices in four key areas: vaccination data, allocation and distribution, access, and community outreach and communication. Within the checklist, each focus area is presented with an overarching statement defining its fundamental purpose.

For example, the focus area of “vaccine data” is delineated as the need for distributors to “Provide real-time data regarding vaccine access and uptake to monitor and address vaccine inequities.” Furthermore, the focus area of “access” is defined as the need to “Reduce barriers and provide the necessary resources for communities to achieve equitable access to the COVID-19 vaccine.”

To make each focus area's overarching goal actionable, we provide four to five explicit and measurable items pivotal to equitable development and implementation of vaccine distribution programs at each level of the social ecological model.^51–53^

For example, historically rooted exclusion from and mistrust of medical and public health systems by members of minority and immigrant populations^12,14,54,55^ was addressed in checklist items pertaining to community outreach and communication and vaccine data. Specifically, “Communicate messages that promote anti-racism, anti-xenophobia, and anti-hate as it relates to COVID-19. Act on this messaging by providing/identifying methods to report transgressions and include this information” and “Make vaccine distribution data readily available and comprehensible to members of the community.”

Policy- and community-level barriers are addressed in the item, “Create multilingual, culturally sensitive, and literacy-appropriate education and outreach strategies available across multiple media platforms and discussion formats designed to reach the most impacted communities.” Although the underlying issues at the root of inequities exacerbated by COVID-19 cannot be solved by performing the checklist items, we aimed to provide recommendations for actions that could be implemented swiftly to mitigate the ongoing inequitable outcomes of vaccine distribution in OC.

**FIG. 2.**
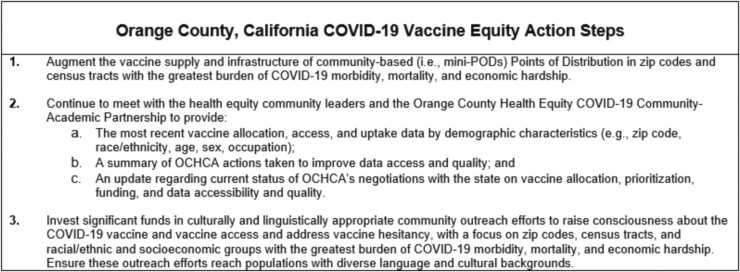
Orange County, California COVID-19 vaccine equity action steps.

To further strengthen the focus on vaccine equity, we prioritized three action steps grounded in local contextual considerations, community partner recommendations, and best practices emerging in the literature (Fig. 2).

These action steps sought to address the fundamental issues shaping barriers to equitable vaccine implementation, including the need to (1) implement an evidence-based vaccine distribution strategy that prioritizes the most affected social groups by applying an intersectional approach (e.g., vaccinate elders in census tracts with highest COVID-19 morbidity and mortality rates); (2) engage in authentic partnership with local community leaders and improve transparency; and (3) engage in contextually, culturally, and linguistically appropriate outreach efforts to combat mistrust in science and public health institutions that have escalated in the pandemic and have been further galvanized by inequitable vaccine rollout and shutting out of information streams.

To put these tools into action, we combined the checklist, a written explanation of its goals and origins, and the action steps into a memo. After feedback and iterative review by community partners followed by academic partners, partnership members were invited to endorse the memo as signatories. After careful discussion with community partners, they requested that the memo be sent on behalf of the partnership and signed by academic partners to support local community-based organizations that navigate a delicate role of advocating for change with a powerful institution that shapes the distribution of county resources. In February 2020, we sent the memo to OCHCA and the county board of supervisors with the request for continued communication regarding plans to enact the outlined distribution practices.

### Continued adaptation of vaccine equity best practices

As development of the checklist, action steps, and memo was underway, partnership members discussed balancing the significance of completeness of the vaccine equity requests versus urgency of delivering the checklist and memo to shape vaccine equity locally. The concern was that too much weight would be placed on perfecting the tool, taking away from the timeliness of its delivery. To maximize potential impact, partnership members collectively determined that the memo should be produced in a manner that prioritized prompt distribution. With this decision came the understanding that the issues addressed, and recommendations made will need to be updated over time as equity concerns evolve and local practice-based evidence expands.

## Discussion

Equitable COVID-19 vaccine distribution and uptake are pivotal to successfully reducing COVID-19 morbidity, mortality, and associated inequities.^4,13^ Without increased attention to best practices for equity across the areas of vaccine data, allocation and distribution, access, and community outreach and communication, communities of color, indigenous communities, and low-income communities will continue to suffer disproportionate negative outcomes.

Purposefully designed to support these needs, the OC COVID-19 vaccine equity best practices checklist demonstrates how vaccine equity best practices can be communicated and promoted across various stakeholder groups throughout the development, mobilization, and continued adaptation of vaccine programs. Moreover, our partnership's nascency, goals, and actions to date serve as a practical demonstration regarding authentic community–academic–practice relationships focused on health equity.

### Responding to continually changing COVID-19 vaccine equity landscapes

Following the initial composition of the OC COVID-19 vaccine equity best practices checklist, California shifted to a new distribution scheme, with a major insurer managing state-wide distribution efforts—a role that lasted only a few months.^56–58^ Under this coordination, it stood likely that although many of the crucial equity considerations would remain the same, the approach required to appropriately advocate for and address equity concerns would change.

Notably, authentic relationships and our practice of continual one-on-one and meeting-based check-ins among partnership members helped to further surface an underlying critical analysis of the fact that, to date, our local multicounty serving entities (e.g., hospitals and academic medical centers) have escaped our focus on vaccine equity advocacy. As contexts continually fluctuate, advocates must be ready and willing to adjust in response to shifts at local, state, and national levels.

### Strengths, future uses, and limitations

A major strength of the checklist comes from the collaborative efforts and input from diverse partnership members, which allowed for a community-centered and locally contextualized evaluation of pressing issues. In addition, our process highlights the ability to create institutionalized opportunities for community partners to be authentically involved in decision-making processes—particularly those related to advancing equity through acknowledging community-specific needs. To this end, we understand that each municipality, county, or state effort to achieve equitable vaccination faces diverse challenges, thus we do not anticipate the content of this tool to be uniformly appropriate across diverse settings. Yet, the actions outlined here may prove a useful technique in advocating for change.

In addition, we recognize that partnerships that equitably bring together community, academic, and public health practice partners have the potential to increase understanding of how racial, social, and economic inequities unfold locally and develop and implement opportunities for structural intervention.^59–61^ Other partnerships see these same COVID-19 equity opportunities and have formed or shifted their focus with an aim to address similar challenges.^62,63^

Although we are acutely aware that the development and distribution of our checklist do not solve the systemic barriers responsible for COVID-19 inequities at their root, our partnership and this advocacy tool were a decisive response to the prevailing emergency environment that unevenly impacted historically marginalized communities. In addition to advocating for equity when addressing the immediate threats posed by inequitable vaccine distribution in OC, our partnership aims to broaden its scope of advocacy efforts to address structural drivers of health inequities in OC. The equitable relationship between community, academic, and practice partnership members is pivotal to achieving this goal.

Furthermore, as was discussed in several of our partnership meetings, community–academic–practice partnerships are critical to amplifying the voices of community-based organizations who are otherwise vulnerable given often delicate relationships based on inequitable power relationships between affected communities and governmental institutions. Utilizing the less vulnerable positionality of universities to advocate for community-identified priorities is essential to advocating for equity until authentic partnerships between community-based organizations and governing institutions can be achieved.

In addition, partnerships or coalitions that bring together representatives of multiple affected communities can transform common dynamics created by racial capitalism, which often segregate issues and position organizations and initiatives to compete against each other for perceived scarce resources and thus impede collective action. By providing a space that supports and connects diverse community-based organizations and initiatives through inclusive conversations regarding shared and disparate needs, community–academic–practice partnerships can strengthen relationships across organizations, initiatives, and groups; foster a collective identity; and advance mutual equity initiatives such as the OC COVID-19 vaccine equity best practices checklist.^59–61^

### Health equity implications

Health inequities effecting low-income communities, communities of color, and indigenous communities across the United States as a result of the COVID-19 pandemic will persist if vaccines are not equitably allocated, distributed, and accepted within the most impacted communities.^15^ Officials in charge of COVID-19 vaccine distribution plans should aim to engage in authentic and equitable partnerships with community-based organizations and coalitions to gain insight into locally contextualized community needs.

Our partnership provides an example of an approach to effectively integrate these groups into critical dialogue and strategic planning regarding each element of an equitable vaccine strategy. Partnerships such as ours are not only necessary within the immediate landscape of COVID-19 vaccinations but will also need to continue so that we can understand and address the structural changes necessary for the reduction of both existing and forthcoming health inequities.

## Conclusion

The COVID-19 pandemic has had disproportionate negative social, economic, and health-related impacts on various marginalized communities throughout the United States.^1–9^ These effects relate to risk of exposure, limited access to resources to protect against transmission, and related morbidity and mortality—all of which are grounded in longstanding racial, social, and economic inequities.^1–9^ Although COVID-19 vaccinations have become more widely available, systemic and organizational barriers have furthered disproportionate impacts through the inequitable distribution, prioritization, and uptake of vaccines.^1–9,64^

Authentic partnerships between community-based organizations and initiatives, academic institutions, and public health practitioners are essential to understanding and addressing structural change needed to reduce and eventually eliminate inequities. Our partnership developed the OC COVID-19 vaccine equity best practices checklist to guide a more equitable vaccine approach with an emphasis on the pivotal role community-based knowledge and localized context should have in shaping recommendations.

This checklist was developed to provide guidance within the immediate emergency setting focusing on local public health actions that were not being implemented within OC. Similar collective approaches and advocacy will be necessary as we face continued COVID-19 vaccine distribution, booster vaccinations, and future pandemic events.

## Abbreviations Used

COVID-19: coronavirus disease 2019
OC: Orange County, California
OCHCA: Orange County Health Care Agency
POD: points of distribution
SARS-CoV-2: severe acute respiratory syndrome coronavirus 2
